# Machine learning reveals sex differences in distinguishing between conduct-disordered and neurotypical youth based on emotion processing dysfunction

**DOI:** 10.1186/s12888-025-06536-6

**Published:** 2025-02-06

**Authors:** Gregor Kohls, Erik M. Elster, Peter Tino, Graeme Fairchild, Christina Stadler, Arne Popma, Christine M. Freitag, Stephane A. De Brito, Kerstin Konrad, Ruth Pauli

**Affiliations:** 1 Department of Child and Adolescent Psychiatry, Faculty of Medicine, TUD Dresden University of Technology, German Center for Child and Adolescent Health (DZKJ), partner site Leipzig/Dresden, Dresden, Germany; 2 School of Computer Science, University of Birmingham, Birmingham, UK; 3 Department of Psychology, University of Bath, Bath, UK; 4 Department of Child and Adolescent Psychiatry, Psychiatric University Hospital, University of Basel, Basel, Switzerland; 5 Department of Child and Adolescent Psychiatry, VU University Medical Center, Amsterdam, the Netherlands; 6 Department of Child and Adolescent Psychiatry, Psychosomatics and Psychotherapy, University Hospital Frankfurt, Goethe University, Frankfurt am Main, Germany; 7 Centre for Human Brain Health, School of Psychology, University of Birmingham, Birmingham, UK; 8 Child Neuropsychology Section, Department of Child and Adolescent Psychiatry, Psychosomatics and Psychotherapy, RWTH Aachen University, Aachen, Germany; 9 JARA-Brain Institute II, Molecular Neuroscience and Neuroimaging, RWTH Aachen & Research Centre Juelich, Juelich, Germany

**Keywords:** Conduct disorder, Emotion processing, Emotion dysfunction, Machine learning, Sex differences, Youth, FemNAT-CD

## Abstract

**Background:**

Theoretical models of conduct disorder (CD) highlight that deficits in emotion recognition, learning, and regulation play a pivotal role in CD etiology. With CD being more prevalent in boys than girls, various theories aim to explain this sex difference. The “differential threshold” hypothesis suggests greater emotion dysfunction in conduct-disordered girls than boys, but previous research using conventional statistical analyses has failed to support this hypothesis. Here, we used novel analytic techniques such as machine learning (ML) to uncover potentially sex-specific differences in emotion dysfunction among girls and boys with CD compared to their neurotypical peers.

**Methods:**

Multi-site data from 542 youth with CD and 710 neurotypical controls (64% girls, 9–18 years) who completed emotion recognition, learning, and regulation tasks were analyzed using a multivariate ML classifier to distinguish between youth with CD and controls separately by sex.

**Results:**

Both female and male ML classifiers accurately predicted (above chance level) individual CD status based solely on the neurocognitive features of emotion dysfunction. Notably, the female classifier outperformed the male classifier in identifying individuals with CD. However, the classification and identification performance of both classifiers was below the clinically relevant 80% accuracy threshold (although they still provided relatively fair and realistic estimates of ~ 60% classification performance), probably due to the substantial neurocognitive heterogeneity within such a large and diverse, multi-site sample of youth with CD (and neurotypical controls).

**Conclusions:**

These findings confirm the close association between emotion dysfunction and CD in both sexes, with a stronger association observed in affected girls, which aligns with the “differential threshold” hypothesis. However, the data also underscore the heterogeneity of CD, namely that only a subset of those affected are likely to have emotion dysfunction and that other neurocognitive domains (not tested here) probably also contribute to CD etiology.

**Clinical trial number:**

Not applicable.

**Supplementary Information:**

The online version contains supplementary material available at 10.1186/s12888-025-06536-6.

## Background

Conduct disorder (CD) is one of the most prevalent mental disorders in children and adolescents, involving severe and repeated antisocial and aggressive behaviors [1]. Although CD is less prevalent and often emerges later in girls than in boys, it is still one of the most commonly diagnosed disorders in both female and male youth [2]. In recent decades, CD diagnoses have increased significantly in both sexes in Western industrialized countries [3], and CD is now one of the most common reasons for referral of youths to medical and youth welfare services, at enormous cost to healthcare services and society in general [4]. Paradoxically, though, CD is one of the least studied, funded, and understood mental disorders in youth– with a particular dearth of research on girls [5].

In particular, the neurocognitive mechanisms that contribute to the emergence and maintenance of CD-related behaviors, such as aggression, remain poorly understood [5], although they may differ– at least in part– by sex [6, 7]. However, research on the mechanisms underlying CD has traditionally focused primarily on boys (or occasionally on small samples of girls only), with studies including mixed-sex samples generally underpowered to detect sex-by-group interaction effects. As part of the FemNAT-CD consortium [2], we have begun to systematically address the under-representation of girls with CD in research by studying the largest representative sample to date of more than 1600 youth with and without CD (~ 65% girls) [8]. This includes neurocognitive assessments of emotion processing skills in a large proportion of them (*N* > 1200) [9, 10]. Mounting evidence suggests that dysfunction in different emotion skill domains, such as emotion recognition (e.g. difficulty identifying facial expressions), emotion learning (e.g. difficulty learning from punishment cues), and emotion regulation (e.g. difficulty inhibiting impulsive responses to emotional cues) may provide a particularly strong basis for explaining possible sex differences in CD [11]. Moreover, recent neurocognitive models of CD etiology emphasize that dysfunction in the three emotion skill domains mentioned above may be associated with different pathways and manifestations of CD in affected youth, particularly related to established DSM-5 subtypes of CD (i.e. with vs. without Limited Prosocial Emotions (LPE)– also known as Callous-Unemotional (CU) traits, including reduced guilt and empathy, callousness, and uncaring attitudes) [12]. It is however still unclear to what extent this applies to both conduct-disordered boys and girls, or whether more female-tailored neurocognitive accounts are needed to explain the origins of CD (and its subtypes) in girls [13].

In general, girls perform better on emotion processing tasks than boys, indicating superior emotion processing skills [14]. This female advantage is already evident in early childhood and continues into adolescence, and may be due to earlier maturation of brain circuits involved in emotion processing [7]. In addition, girls are traditionally discouraged from engaging in antisocial behavior by society (including parents, peers and teachers) to a much greater extent than boys [15]. Thus, in order to overcome these ‘advantageous’ factors (i.e. to cross the higher threshold into psychopathology) and develop CD, it has been hypothesized that girls require greater vulnerability– that is, a combination of more harmful dispositional and environmental risk factors including neurocognitive dysfunction– to develop the disorder [16].

We recently tested this “differential threshold” hypothesis of female CD with respect to emotion functioning in a well-powered and thoroughly clinically assessed sample of girls and boys with CD (*n* = 542) and neurotypical controls (*n* = 710) from the FemNAT-CD cohort [9, 10]. More specifically, we explored the prediction that girls with CD would have more pronounced emotion dysfunction relative to neurotypical girls than boys with CD by using a neurocognitive test battery that covered emotion recognition, learning, and regulation skills. However, when applying traditional mass-univariate statistical analyses to test for sex-by-group interaction effects, we were unable to support the “differential threshold” hypothesis: Girls with CD did not show more pronounced neurocognitive dysfunction than boys with CD in any emotion domain. On the contrary, for emotion recognition, we even found that boys with CD were 2.1 times more likely to have a clinically-meaningful dysfunction (i.e. task performance within the bottom 10% of their age- and sex-matched neurotypical peers) than girls with CD [9, 10].

Overall, the available literature provides a rather mixed– and in part even contradictory– picture of possible sex differences in the emotion functioning of youth with CD (for a brief overview, see [9]). Clearly, further research efforts are needed to better disentangle sex-specific (if present) from sex-unspecific neurocognitive domains that contribute to CD. This may require novel analytic approaches that go beyond the traditional ones typically used in the field. Although conventional statistical procedures are useful for examining mean differences in task performance at the group level by using an explicit statistical model to test a particular hypothesis, these typically (mass) univariate techniques tend to ignore known critical interactions between variables and rarely provide meaningful measures of predictive accuracy at the individual level [17]. A machine learning (ML) classifier has the advantage of being able to determine the accuracy with which conduct-disordered girls and boys and their neurotypical peers can be differentiated from each other on a multivariate basis (i.e. all emotion processing variables across all neurocognitive tasks are considered simultaneously), coupled with its capacity to make predictions about individuals on their class membership. The classifier can therefore quantify how reliable differences in each emotion domain are as markers of CD in individual female and male youths. In the current study, we used Angle-based Generalized Matrix Learning Vector Quantization (Angle-GMLVQ), a prototypical multivariate, supervised ML classifier technique (see [18] for more details; see also the Methods section below). It has an integrated feature relevance scoring or ranking procedure that quantifies the importance of each feature (i.e. task performance variable) to the classification decision in the context of multiple parallel features. Angle-GMLVQ captures the relative differences (angles) between the features rather than their absolute magnitude, which makes it particularly sensitive to different patterns of task performance in the three emotion domains tested (e.g. difficulty processing punishment cues but not certain facial expressions). An example of this approach can be found in [19], where we used Angle-GMLVQ to classify CD with high CU traits, CD with low CU traits, and neurotypical controls based solely on differences in emotion recognition skills, without a specific focus on sex-related effects. Importantly, although Angle-GLMLVQ is a relatively new ML classification technique, it has been repeatedly shown to perform as well as more established classifiers such as support vector machines (SVM) [19, 20] or even to offer some advantages over, for example, SVM-based methods (for details, see [21]).

For the current study, we reanalyzed the neurocognitive task performance data from the FemNAT-CD multi-site cohort (*N* = 1252), for which data from all three emotion skill domains (i.e. all three tasks) were fully available. It was the same sample that we previously investigated using conventional statistical methods in [9, 10]. Thus, the primary aim here was to re-examine this dataset using Angle-GMLVQ as the ML classifier of choice. In doing so, we sought to determine the extent to which emotion processing dysfunction is a different (or similar) predictor of CD in individual girls and boys with this disorder compared to neurotypical controls, and which emotion domain– and therein which neurocognitive features– contribute most to such predictions. Although Angle-GMLVQ classification is a data-driven rather than a hypothesis-driven approach, we had several assumptions based on (our) earlier findings: First, given the previously established case-control differences in neurocognitive task performance in all three emotion skill domains, in which girls and boys with CD showed similar deficits, we predicted that classifier performance would be above chance level (i.e. >50% accuracy) when differentiating between CD girls and neurotypical girls and between CD boys and neurotypical boys; a fair and realistic estimate would be between 60 and 65% classification accuracy, considering related studies in the field of attention deficit hyperactivity disorder (ADHD; e.g [22, 23]). Since we did not find sex-by-group interaction effects, one would also expect the performance of the two classification models to be comparable. In contrast, however, using a data-driven ML algorithm may lead to different, more accurate results [17], with one classifier being superior to the other. This would be the case if CD girls and CD boys, compared to their respective neurotypical peer groups, are characterized by different patterns of difficulty in emotion processing skills (i.e. amount and/or types of neurocognitive features that distinguish cases from control groups vary significantly between the sexes). In this context, we expected that some neurocognitive features from the three emotion domains would be particularly relevant as predictive markers for female CD and some for male CD, although no clear predictions were made due to the inconsistency of the available literature.

## Methods

### Participants

For this study, we reanalyzed the neurocognitive data of 542 youths with CD (317 girls) and 710 neurotypical controls (479 girls), aged 9–18 years, from the FemNAT-CD multisite project (see [9, 10] for more details on participant recruitment, assessment battery, and phenotypic characteristics). Participants were included if they provided a complete set of data from the test battery, covering emotion recognition, emotion learning, and emotion regulation skills (see below). Participants were recruited from the community (e.g. mainstream schools) as well as psychiatric hospitals, social welfare facilities and offending services for youth in different locations across seven European countries (Table S1). Exclusion criteria for youth with CD and neurotypical controls included IQ < 70, autism spectrum disorders, schizophrenia, bipolar disorder or mania, neurological disorders, and genetic syndromes. Individuals with CD had to fulfill the current diagnosis for CD according to DSM-IV-TR criteria [24]. Neurotypical controls had to be free of current psychiatric disorders and a history of CD, ODD, and ADHD. All current and lifetime psychiatric diagnoses (incl. the respective DSM-IV-TR subtypes, age-of-onset, duration, and severity of the disorder) were determined by trained staff using the Kiddie-Schedule for Affective Disorders and Schizophrenia–Present and Lifetime version (K-SADS-PL [25]), a semi-structured clinical interview administered separately to participants and their caregivers. Total IQ were estimated with the Wechsler Intelligence Scales [26–28]. A proxy for the LPE specifier for CD (according to DSM-5; see [29]) were derived from the self-report Youth Psychopathic traits Inventory (YPI; i.e. the subscales “remorselessness”, “unemotionality”, and “callousness”) [30]. Socioeconomic status (SES) was estimated based on parental income, education level, and occupation, normalized for the participant’s country [20]. See [9, 10] for psychometric information on these measures. Table S2 summarizes the sample’s main demographic and clinical characteristics.

### Neurocognitive test battery

We used three neurocognitive tasks to assess emotion recognition (i.e. *Emotion Hexagon task*), emotion learning (i.e. *Passive Avoidance Learning task*), and emotion regulation skills (i.e. *Emotional Go/Nogo task*) in the FemNAT-CD cohort (for details, see [9, 10]). We selected this test battery based on influential models of emotion dysfunction in CD, and because the tasks are widely used in neurocognitive research on emotion functioning to distinguish between clinical groups (incl. CD, ADHD, and internalizing disorders) and neurotypical controls [12]. In addition, the available psychometric information supports both the validity and reliability of the three tasks [9]. For example, the extracted performance (i.e. dependent) variables per task that we examined previously and in the current study (Table S3) had acceptable to good internal consistencies (Cronbach’s *α* ≥ 0.70), and correlations between these variables were greater within each of the three emotion skill domains (*r*_mean_=0.37, 95% CI: 0.34, 0.44) than between the domains (*r*_mean_=0.12, 95% CI: 0.07, 0.17; Fisher’s *z* = 7.28, *p* <.001), suggesting that the test battery did indeed capture emotion functioning as a multifaceted construct [10].

In short, we administered the *Emotion Hexagon task* to assess emotion recognition skills, including the six basic facial expressions happiness, sadness, anger, fear, disgust, and surprise (incorrect recognition per expression was counted as errors in %). We administered the *Passive Avoidance Learning task* to assess emotion learning in the form of assigning reward or punishment values to previously unknown stimuli. In this task, participants had to learn by trial-and-error to respond to four stimuli eliciting rewards (gaining 1, 700, 1400, or 2000 points, respectively; non-responses were counted as omission errors in %) and to avoid responding to four stimuli eliciting punishments (losing 1, 700, 1400, or 2000 points, respectively; responses to these stimuli were counted as avoidance errors in %). Finally, we administered the *Emotional Go/Nogo task* to assess emotion regulation, defined as the ability to maintain inhibitory control when confronted with interfering emotional information, including positive or negative facial expressions. In this task, participants had to press a response button as quickly and accurately as possible to a specified facial expression (i.e. the go stimuli) and inhibit responding to any other facial expression (i.e. the nogo stimuli; responses to these stimuli were counted as false alarm errors in %). Six blocks of go/nogo pairings were randomly presented: neutral-happy, neutral-fearful, happy-neutral, fearful-neutral, happy-fearful, and fearful-happy. More details on the test battery and procedures are provided in the supplement (see also [9, 10]).

### ML classification and feature relevance scoring

First, because covariates cannot be controlled for in ML classification analyses, all 20 raw performance scores-of-interest from our test battery (Table S3) were age-, IQ-, SES-, and site-adjusted (given the case-control differences in these variables) using standard regression procedures, resulting in *z*-scores as the dependent variables in subsequent analyses. In order to assess ML classification performance, two Angle-GMLVQ models were created separately for the two sexes using the *z*-scores as features (i.e. predictor variables). Classes were (i) CD girls against neurotypical girls in the female model, and (ii) CD boys against neurotypical boys in the male model. By projecting the neurocognitive data into a multidimensional feature space where each feature represents a different dimension, a classifier can construct a decision boundary that optimally separates individuals of the two different classes within this space. In doing so, it considers all features and their interactions simultaneously. This decision boundary is then used to predict the class membership of previously unseen individuals based on their position in the feature space (e.g. the membership of an individual to the female CD group or the female neurotypical control group).

For each of the two models, 500 separate classifiers were repeatedly trained and tested using data re-sampling to ensure stability of each model (see the supplement for a full description of the training and testing procedure using a holdout design). After the models were fully trained and tested, mean performance metrics for the female and male models were calculated separately and then compared (see below). Model performance was evaluated using macro-averaged, weighted accuracy (wACC; based on [21]). This is the mean of the accuracies for each class per model, accounting for imbalanced class sizes. Per model, we also calculated the (i) positive predictive value (PPV; i.e. the proportion of participants classified as ‘CD’ who were actually CD), (ii) negative predictive value (NPV; i.e. the proportion of participants classified as ‘neurotypical controls’ who were actually neurotypical controls), true positive rate (TPR = sensitivity; the proportion of CD participants who were classified correctly), and true negative rate (TNR = specificity; the proportion of neurotypical participants who were classified correctly). We statistically compared these classification measures between female and male models according to the recommendations of [31] (but see also [32]).

For each trained classifier, the Angle-GMLVQ algorithm generates a feature relevance score for each neurocognitive feature. The relevance score of a feature is a non-negative number that quantifies the importance of that feature for the respective classification task. The relevance scores are normalized to sum to 1 across all features in the model. This is done to stabilize subspace learning employed in the Angle-GMLVQ algorithm, but it also allows the relevance scores learned in the 500 classifiers to be directly compared. The procedure described above thus generates 500 feature relevance scores for each feature per model. However, some of these classifiers fail to distinguish between the groups (i.e. they do not achieve at least 50% macro-averaged accuracy), and the feature relevance scores from these classifiers are therefore not informative and are discarded (retained classifiers: female model *n* = 493, male model *n* = 476). We emphasize that the inferior models are discarded based on their in-sample (training) performance so that there is no information leakage from the out-of-sample (test) data. We note that such a feature score learning algorithm is effectively performing feature selection and feature ranking [33]. For each model, we then calculated the percentage of resamplings in which a neurocognitive feature was among the five top-scoring features that best differentiated between CD and neurotypical controls with a macro-averaged, weighted accuracy (wACC) of ≥ 0.6 (i.e. macro-averaged classification error rate ≤ 0.4; see below).

## Results

### Classifier performance

Angle-GMLVQ model performance is shown in Table 1. First, we compared the classification performance for the female and male models (wACCs). As expected, both models performed significantly above chance level (i.e. >50%; binominal tests: *p*s < 0.001) with large effect sizes (Cohen’s *g*s ≥ 0.45), but the overall classification performance was below the clinically relevant threshold of 80% accuracy [34]. Nevertheless, the classifier performance still provided a relatively fair and realistic estimate that can be achieved in a large, heterogeneous, multi-site sample based on neurocognitive variables alone (see for similar findings relevant studies in the field of ADHD e.g [22, 23, 35]). Notably, the female model significantly outperformed the male model (see wACCs in Table 1). Moreover, both models did not differ in their ability to correctly identify neurotypical controls (TNRs), but the female model was significantly better at identifying youths with CD than the male model (TPRs).

**Table 1:** Model performance of the Angle-GMLVQ classifier

	Female model: CD girls vs. neurotypical girls	Male model: CD boys vs. neurotypical boys	Test statistics
	Mean [95% CIs]		*p*s, *r*s
Weighted Classification Accuracy (wACC)	0.59 [0.58, 0.59]	0.56 [0.55, 0.56]	**< 0.001**, 0.34
Positive Predictive Value (PPV)	0.59 [0.58, 0.59]	0.46 [0.46, 0.47]	**< 0.001**, 0.83
Negative Predictive Value (NPV)	0.59 [0.59, 0.59]	0.65 [0.65, 0.65]	**< 0.001**, 0.63
True Positive Rate (TPR = sensitivity)	0.57 [0.57, 0.58]	0.52 [0.51, 0.52]	**< 0.001**, 0.38
True Negative Rate (TNR = specificity)	0.60 [0.59, 0.61]	0.60 [0.59, 0.60]	0.64, 0.02

CD = conduct disorder; CIs = confidence intervals; *p*s = *p*-values based on non-parametric Mann-Whitney-U tests; *r*s = Pearson’s correlation coefficient (where 0.1, 0.3, and 0.5 represent small, medium, and large effects, respectively). Bold *p*-values indicate significant results. We repeated the classification analyses using a support vector machine (SVM) in JASP (version 0.19.2) and found that the pattern of results was similar (i.e. Female model: ACC = 0.60; Male model: ACC = 0.56). It should also be noted that we did not assess the gender identity of the participants, but assigned them to the female or male sample based on their sex at birth

### Feature relevance

**Fig. 1:**
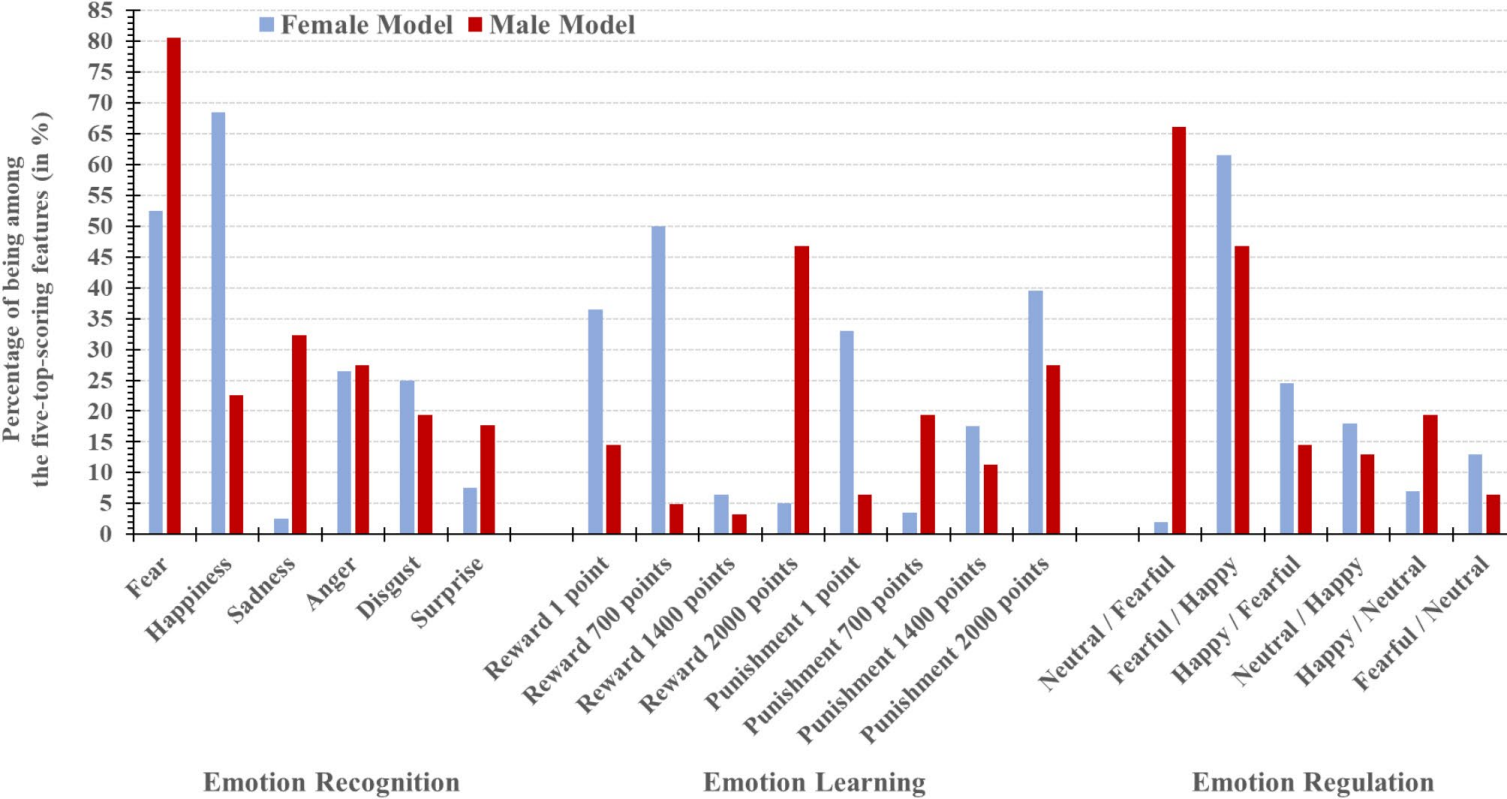
Feature relevance scores for the female model and the male classification model. The bars show the percentage of resamplings in which a neurocognitive feature was among the five top-scoring features that best differentiated between CD and neurotypical controls with a macro-averaged, weighted accuracy (wACC) of ≥ 0.6 (i.e. macro-averaged classification error rate ≤ 0.4)

Figure 1 shows the relevance scores for each neurocognitive feature per emotion domain separately for the female and male models. When considering the highest-ranking features (i.e. those with the highest percentages) that best differentiated between CD and neurotypical controls, they came from all three emotion domains in both models, but also varied somewhat in type. While recognizing ‘happiness’ appeared to be the most relevant predictor for female CD (i.e. it had the highest percentage of resamplings in which this neurocognitive feature was among the five top-scoring features that best differentiated between CD girls and neurotypical girls with a wACC of ≥ 0.6), recognizing ‘fear’ appeared to be the most relevant predictor for male CD (whereas, for example, the recognition of happy facial expressions was a much less relevant predictor in the male model).

## Discussion

The primary aim of the present ML study was to examine whether dysfunction in three emotion skill domains linked to CD [12] are different or similarly reliable predictors of the disorder in individual girls and boys diagnosed with CD compared to neurotypical controls, and which emotion domain/neurocognitive features best contribute to such a prediction. As expected, the female and male classification models performed above chance level (with large effect sizes) and were thus reliably able to distinguish both female and male individuals with CD from their neurotypical peers on the basis of the multidimensional features of emotion dysfunction. However, but as expected, overall classification results were well below a clinically relevant threshold (i.e. <80% classification accuracy [36])– although, with classification accuracies of approximately 60%, they still provided relatively fair and realistic estimates (e.g [22]). This likely reflects the considerable heterogeneity in emotion functioning within such as large and diverse, multi-site cohort of CD youth (including substantial overlap in neurocognitive task performance with neurotypical controls), as we have previously shown with non-ML statistical analyses of the same dataset [10]. Most interestingly, the female classification model slightly outperformed the male model and was slightly better at identifying individual youths (i.e. girls) with CD, suggesting that the emotion processing difficulties studied here may be a more reliable marker of female CD than male CD. This finding contrasts with our previous work on the same dataset, where we were unable to detect sex differences in emotion recognition, learning, and regulation dysfunction in CD versus neurotypical controls using classical mass-univariate statistical analyses [9, 10]. Most likely, this discrepancy reflects that ML is more powerful than conventional statistics at finding generalizable predictive patterns in the multidomain dataset under consideration [37]. Looking at just the top-ranking, i.e. most discriminative, neurocognitive features in the female and male models, they stem from all three emotion domains. However, they appeared to be both specific and unspecific to each model: For example, while recognizing happy facial expressions was the strongest predictor for female CD, recognizing fear was the strongest predictor for male CD, but was still among the highest-ranking features in the female model.

These new ML results extend findings from previous behavioral studies that have found dysfunction in emotion recognition, learning, and regulation in conduct-disordered girls and boys, most of which have used classical statistical models to test specific hypotheses (including our own work [9]). By combining a theory-driven and data-driven analytic approach, we were able to confirm that the full set of neurocognitive features tested here, spanning multiple emotion skill domains, function as reliable single-subject markers of CD in girls and boys, supporting currently influential models of CD etiology [12]. However, although the accuracy of predicting CD in both girls and boys was well above chance and provided realistic estimates (taking into account relevant studies in the related field of ADHD research [22, 23]), they did not reach a level that would indicate possible clinical utility [34]; note, though, that in this ML context ‘objective’ neurocognitive measures usually perform considerably worse than rating scales or clinical interviews (e.g [35]). Moreover, girls and boys with CD were much more likely to be misclassified than their neurotypical peers, demonstrating that the neurocognitive task performance of many individuals in the two CD groups was more similar to that of neurotypical controls than vice versa. These results were not entirely unexpected and underscore that CD is indeed a remarkably heterogeneous condition [5, 12]. They suggest that emotion dysfunction is certainly more prevalent in female and male CD than in neurotypical controls, but there is a considerable proportion of youth with CD who perform normally in the domains of emotion functioning examined here, which is consistent with other research in this area [19, 38]. Therefore, emotion dysfunction may be etiologically and clinically relevant only for some specific subgroups of girls and boys with CD, but not for the whole population of youth with this diagnosis. Other neurocognitive mechanisms beyond emotion processing likely contribute to the manifestation of CD and should be considered in future ML studies (see e.g [39]).

One could criticize that our study design using ML is suboptimal to thoroughly test the “differential threshold” hypothesis that neurocognitive dysfunction is *more* pronounced in female than in male CD. Notably, it could also be speculated that girls with CD may acquire a greater degree of emotion recognition, learning and regulation due to social pressures and would therefore show *fewer* differences to neurotypical girls than boys. Interestingly, however, the Angle-GMLVQ models created here showed better classification performance and identifiability of individual girls than boys with the diagnosis, with medium to large effect sizes. Thus, emotion dysfunction appears to better predict the presence of CD in affected girls than in boys. This suggests that the pattern of aberrant emotion processing skills tested here may manifest more consistently (i.e. less heterogeneously) in girls than in boys with CD. In this context, we identified several female-specific (e.g. recognition of happy facial expressions) neurocognitive features from all three emotion skill domains that had the greatest impact on the prediction of CD in girls compared to boys. Although we have previously argued, based on results for the same sample using conventional statistics, that neurocognitive models of CD apply equally to both sexes, the current findings suggest that some neurocognitive features may be particularly relevant in female CD.

This study had a number of notable strengths (see also [9, 10]). These include (i) the use of a multivariate ML classifier with integrated feature relevance ranking, thereby avoiding the methodological limitations associated with classical mass-univariate, group-based inferential statistics, which too often produce significant but non-reproducible results [17]; (ii) a large, representative, mixed-sex, and thoroughly clinically assessed cohort of youth with a confirmed diagnosis of CD– thus addressing the under-representation of girls in earlier CD research; and (iii) the administration of a comprehensive neurocognitive test battery that spanned several core, theory-based emotion domains related to CD etiology in order to measure multiple neurocognitive features simultaneously within the same sample.

However, our study also had several limitations (see also [9, 10]): (i) Since this study only included youth aged 9 to 18, the current findings cannot be readily applied to younger children with CD. (ii) It should be noted that we did not perform sensitivity analyses (e.g. we did not ‘control for’ the presence of major comorbidities, such as ADHD), because we did not find any effects of comorbidities on the main outcome variables in our previous studies with the same large dataset and sample, including one ML [19] and two non-ML studies [9, 10]. We therefore assumed that this would also be the case here, which did not justify such analyses [40]. (iii) Ideally, however, we would have validated our ML classifier through further testing with a completely independent dataset. Unfortunately, this was not possible as no such datasets were available. To advance this line of research, it would be beneficial to incorporate additional data sources (e.g. other neurocognitive measures beyond emotion processing and/or phenotypic information other than neurocognitive features) into the same classification model(s) to replicate and extend the results obtained here, thereby substantiating the interpretation and generalization of the current findings. This could also include a comparison of different ML algorithms (including methods capable of measuring and visualizing feature interactions [41]) to better understand which algorithms (and their specifications) are better able to identify specific features for sex-specific diagnostic and therapeutic purposes in youth with CD.

## Conclusions

The present study is one of the few ML studies on neurocognition in the field of CD (e.g [19, 39]). It is the first to combine a theory- and data-driven analysis approach to investigate sex differences in three emotion processing domains closely linked to CD etiology [12]. Indeed, the current results support the notion that neurocognitive dysfunction in the three emotion domains is a reliable predictor of female and male CD at the individual level, although this was slightly more so for girls than for boys with this disorder. Thus, these results partly confirm, but also advance, the findings of our previous work (and that of others) based on conventional non-ML statistics most commonly used in this line of research. Certainly, it would be premature to conclude that sex-specific neurocognitive accounts are needed to better explain CD in girls versus boys (see [42] for a similar conclusion based on other domains, such as reading, memory, and vocabulary). However, the current findings suggest that a pattern of specific neurocognitive features may be more important for the manifestation of CD in individual girls than in boys. Yet, given that the ML algorithm achieved a classification and identification accuracy of about 60% for both girls and boys with CD based on the neurocognitive features alone, our data also confirm that CD is a complex disorder and that, accordingly, emotion dysfunction manifests in a heterogeneous manner and affects only some subgroups but not all youth with CD (in line with [10]). Consequently, future studies should further investigate this important research question by using other ML methods for the purpose of subgrouping at the single-subject level (e.g. normative modeling [43]) to more precisely identify and validate novel neurocognitive subtypes of female and male CD within and beyond emotion functioning. This, in turn, will motivate the development of more targeted, patient-centered neurocognitive interventions to help mitigate essential skill deficits in affected youth and could thus improve the effectiveness of behavioral interventions for conduct problems [44].

## Electronic supplementary material

Below is the link to the electronic supplementary material.

Supplementary Material 1

## Data Availability

Data are available from the corresponding author(s) on reasonable request.

## Abbreviations

Angle-GMLVQ: Angle-based Generalized Matrix Learning Vector Quantization
ADHD: Attention deficit hyperactivity disorder
CD: Conduct disorder
CU: Callous-unemotional (traits)
DSM-5: Diagnostic and statistical manual of mental disorders, Fifth edition
FemNAT-CD: Neurobiology and Treatment of Adolescent Female Conduct Disorder: The Central Role of Emotion Processing
IQ: Intelligence quotient
K-SADS-PL: Kiddie-Schedule for Affective Disorders and Schizophrenia for School-Age Children– Present and Lifetime Version
LPE: Limited prosocial emotions (specifier)
NPV: Negative Predictive Value
ML: Machine learning
ODD: Oppositional defiant disorder
PPV: Positive Predictive Value
SES: Socioeconomic status
SVM: Support vector machine
TNR: True Negative Rate (= specificity)
TPR: True Positive Rate (= sensitivity)
wACC: Weighted Classification Accuracy
YPI: Youth Psychopathic traits Inventory
