# Supplementary material for: Machine learning reveals sex differences in distinguishing between conduct-disordered and neurotypical youth based on emotion processing dysfunction
Source: BMC Psychiatry. 2025 Feb 6;25:105. doi: 10.1186/s12888-025-06536-6 (PMC11800453; doi:10.1186/s12888-025-06536-6)
Supplement: Supplementary file 1 — Supplementary Material 1 [file 12888_2025_6536_MOESM1_ESM.docx]

# Supplementary Materials for “Machine learning reveals sex differences in distinguishing between conduct-disordered and typical youth based on neurocognitive features of emotion dysfunction” by Kohls, Elster et al.

Gregor Kohls*,^1^ Erik M. Elster*,^1^ Peter Tino,^2^ Christina Stadler,^3^ Arne Popma,^4^ Graeme Fairchild,^5^ Christine M. Freitag,^6^ Stephane A. De Brito^7^, Kerstin Konrad,^8, 9^ and Ruth Pauli^7^

^1^ Department of Child and Adolescent Psychiatry, Medical Faculty, TU Dresden, Dresden, Germany

^2^ School of Computer Science, University of Birmingham, Birmingham, United Kingdom

^3^ Department of Child and Adolescent Psychiatry, Psychiatric University Hospital, University of Basel, Basel, Switzerland

^4^ Department of Child and Adolescent Psychiatry, VU University Medical Center, Amsterdam, the Netherlands

^5^ Department of Psychology, University of Bath, United Kingdom

^6^ Department of Child and Adolescent Psychiatry, Psychosomatics and Psychotherapy, University Hospital Frankfurt, Goethe University, Frankfurt am Main, Germany

^7^ Centre for Human Brain Health, School of Psychology, University of Birmingham, Birmingham, United Kingdom

^8^ Child Neuropsychology Section, Department of Child and Adolescent Psychiatry, Psychosomatics and Psychotherapy, RWTH Aachen University, Aachen, Germany

^9^ JARA-Brain Institute II, Molecular Neuroscience and Neuroimaging, RWTH Aachen & Research Centre Juelich, Juelich, Germany

*These authors contributed equally to this work and share first authorship.

**Correspondence to:**

# Gregor Kohls, PhD, Department of Child and Adolescent Psychiatry, Faculty of Medicine, TU Dresden, Fetscherstr. 74, 01307 Dresden, Germany; e-mail: [gregor.kohls@ukdd.de](mailto:gregor.kohls@ukdd.de)

## Differences between included and excluded participants

**Table S1.** Number of included participants per group and sex by country

| **Country** | **Total**  *N* | **CD girls**  *n* | **TDC girls**  *n* | **CD boys**  *n* | **TDC boys**  *n* |
| --- | --- | --- | --- | --- | --- |
| Germany | 477 | 107 | 151 | 106 | 113 |
| United Kingdom | 308 | 43 | 110 | 71 | 84 |
| Spain | 142 | 61 | 59 | 12 | 10 |
| Netherlands | 125 | 37 | 72 | 8 | 8 |
| Greece | 109 | 25 | 45 | 25 | 14 |
| Switzerland | 53 | 23 | 30 | 0 | 0 |
| Hungary | 38 | 21 | 12 | 3 | 2 |
|  | **1252** | **317** | **479** | **225** | **231** |

CD=conduct disorder (~26% community-based, ~74% clinically-referred); TDC=typically developing controls (100% community-based). Among the study sites in each country, all centers are clinical departments for child and adolescent psychiatry (with in- and outpatient services), except for the UK sites which are specialized in research on CD with established recruitment schemes through youth offending services, mainstream schools and referrals from caseworkers. However, even the clinical study sites used recruitment strategies outside their own clinical services, e.g. via youth welfare institutions or special schools, although to a varying degree. Please note, recruitment source information was not available on a person-by-person basis, but was estimated based on available recruitment data from subsamples at each site per country.

Prior to data analyses, we excluded 17% of youth with CD and 7% of TDCs with incomplete neurocognitive data. The reasons for missing data were (i) technical difficulties (e.g. software problems), (ii) experimenter errors, (iii) lack of time, (iv) noncompliance, or (v) study dropout. We assume that the incomplete neurocognitive data are missing at random due to the various reasons listed above. Importantly, when comparing the excluded youth with CD with the included youth with CD, we did not find significant differences in age, estimated IQ, SES, psychiatric comorbidity patterns, CD symptomatology, or LPE specifier (all *p*s >.05), suggesting that the reported data for CD are not biased by excluding participants with incomplete datasets.

**Table S2** Selected sample demographics and clinical characteristics per group and sex

|  | **CD girls**  (*n*=317) | **TDC girls**  (*n*=479) | **CD boys**  (*n*=225) | **TDC boys**  (*n*=231) | **Group effect**  *p* value, effect size |
| --- | --- | --- | --- | --- | --- |
| Age (years) *M* (SD) | 14.7 (2.1)^a^ | 14.0 (2.5)^b^ | 13.9 (2.4)^b^ | 13.8 (2.5)^b^ | **<.001**, *η^2^_p_*=.021 |
| Estimated IQ *M* (SD) | 93.9 (12.1)^a^ | 102.9 (12.5)^b^ | 96.3 (12.5)^a^ | 104.7 (11.7)^b^ | **<.001**, *η^2^_p_*=.12 |
| LPE specifier proxy (%) | 38.5^a^ | 15.5^b^ | 51.1^c^ | 24.2^d^ | **<.001**, *Φ*=.30 |
| SES *M* (SD) | -0.30^a^ | 0.22^b^ | -0.28^a^ | 0.39^b^ | **<.001**, *η^2^_p_*=.08 |
| ODD (%) | 76.7 | N/A | 79.6 | N/A | .42, *Φ*=.034 |
| ADHD (%) | 30.0 | N/A | 46.7 | N/A | **<.001**, *Φ*=.17 |
| SUD (%) | 19.2 | N/A | 15.6 | N/A | .27, *Φ*=.048 |
| MDD (%) | 18.8 | N/A | 10.7 | N/A | .08, *Φ*=.11 |
| GAD (%) | 3.8 | N/A | 2.2 | N/A | .23, *Φ*=.10 |

Diagnoses were based on the Schedule for Affective Disorders and Schizophrenia for School-Age Children–Present and Lifetime version (K-SADS-PL). For TDC, any current psychiatric diagnosis as well as a history of ADHD, ODD, or CD was exclusionary. ADHD=attention deficit hyperactivity disorder; CD=conduct disorder; GAD=generalized anxiety disorder; IQ=estimated intelligence quotient; LPE=limited prosocial emotions; MDD=major depressive disorder; Meds=on psychotropic medications; ODD=oppositional defiant disorder; SES=socioeconomic status; SUD=substance use disorder; TDC=typically developing controls. *p* values are based on *F* tests or *Χ*^2^ tests. Significant results are indicated by bold *p* values. Groups with different superscript indices differ significantly in post-hoc comparisons (*p*<.05, Bonferroni corrected). Information on race and/or ethnicity was not collected in accordance with governmental and ethical guidelines in Germany.

## Details on the neurocognitive test battery and testing procedure

We used the *Emotion Hexagon task* to assess the accuracy of facial emotion recognition [1]. Participants were asked to label morphed facial expressions as either happy, sad, angry, fearful, disgusted, or surprised (i.e., the six ‘basic’ emotions). Morphs were created from six expression pairings: happy-surprised, surprised-fearful, fearful-sad, sad-disgusted, disgusted-angry, and angry-happy. Each pair included two prototype expressions in proportions 90:10, 70:30, 50:50, 30:70, and 10:90 (i.e., 10% happy and 90% surprised for the happy-surprised continuum). Morphed expressions were presented individually and randomly on a computer monitor for a maximum of 3 seconds, and participants were asked to select by mouse-click one of the six emotion labels that best described the expression shown. Participants were given as long as necessary to make their selection and were not given feedback about their performance accuracy. Participants completed one practice block, followed by five blocks that each displayed all 30 morphed expressions once (6 pairs x 5 morphs). The total score for incorrect recognition per expression ranged from 20 (100% error rate) to 0 (0% error rate), with 50:50 morphs not being scored or analyzed.

We administered a modified *Passive Avoidance Learning task* to assess the accuracy of emotion learning [2]. The task involves assigning reward and punishment values to novel stimuli (“ziggerins” [3]). Novel stimuli were chosen to tap into pure learning effects without the bias of stimulus familiarity. Participants were instructed to learn by trial-and-error to respond through button press to four different reward stimuli (gaining 1, 700, 1400, or 2000 points, respectively; non-responses were counted as omission errors in %) and to avoid responding to four different punishment stimuli (losing 1, 700, 1400, or 2000 points, respectively; responses to these stimuli were counted as avoidance errors in %). Each stimulus was shown once within a block of 8 trials, with 10 blocks overall (including one practice block). Stimuli were displayed on a computer monitor for a maximum of 3 sec, followed by performance feedback (i.e., amount of points won, or lost, as well as the running total points). Participants started the task with 10,000 points.

We administered the *Emotional Go/Nogo task* to assess the accuracy of emotion regulation defined as the ability to maintain cognitive control when confronted with interfering emotional information, including positive and negative facial expressions [4], [5]. Participants were instructed to press a response button as quickly and accurately as possible whenever a named facial expression appeared on the screen (go trials) and not to press for any other expression (nogo trials). The task included six randomly presented blocks of go-nogo pairings: neutral-happy, neutral-fearful, happy-neutral, fearful-neutral, happy-fearful, and fearful-happy. Each block included 35 go (73%) and 13 nogo (27%) stimuli. The go trials occurred more frequently in order to create a pre-potent tendency for the participant to respond. Stimuli consisted of gray-scaled fearful, happy, and neutral expressions from six male and six female adults, with four African-American, Asian, and Caucasian individuals for each expression type respectively (NimStim set numbers: 6, 8, 11, 14, 15, 16, 27, 36, 39, 43, 44, and 45). Stimulus duration was 500 msec with 1-sec interstimulus intervals. False alarm error rates in % for nogo trials indexed emotion regulation, with higher rates reflecting worse performance [6].

Order of tasks was pseudorandomized separately across group (CD, TDC), sex (girls, boys), and age brackets (9-12, 13-15, and 16-18 years). The extracted performance variables of the three tasks (see Table 2 in the main text) had acceptable to good reliabilities (Cronbach’s *α* ≥ .70). We chose this particular test battery based on influential models of emotion dysfunction in CD (see [7] for details), and because the three tasks have widely been used in neuropsychological research of emotion functioning in developmental psychopathology, including CD, ADHD, and internalizing disorders. Thus, the validity of the test battery comes from its proven usefulness to distinguish between clinical groups and controls in previous research [8]. Available psychometric data further support both reliability and validity of all three neuropsychological measures [6], [9], [10]. Standard operating procedures (SOP) ensured consistency of data collection, handling, and analysis across all data collecting sites.

**Table S3.** Main neurocognitive features per emotion skill domain (and task)

| **Emotion recognition**  **(Hexagon)** | **Emotion learning**  **(Avoidance)** | **Emotion regulation**  **(Go/Nogo)** |
| --- | --- | --- |
| *Error rate in % per facial expression:*   - Happiness - Sadness - Anger - Fear - Disgust - Surprise | *Error rate in % per reward and punishment condition:*   - Reward 1 point - Reward 700 points - Reward 1400 points - Reward 2000 points - Punishment 1 point - Punishment 700 points - Punishment 1400 points - Punishment 2000 points | *False alarm error rate in % per go/nogo pairing:*   - Neutral/Happy - Neutral/Fearful - Happy/Neutral - Fearful/Neutral - Happy/Fearful - Fearful/Happy |

## Angle-GMLVQ model training and testing procedure

The procedure for training and testing each of the individual classifiers per model followed a holdout design with an 80/20 training/testing split, repeated for 500 random sub-samplings in order to ensure stability of the model: First, the data were split into a training set (80% of the data) and a testing set (20% of the data). This was a stratified split, i.e. preserving the original class proportions in both the training and testing sets. The class sizes in the training set were then equalized by randomly down-sampling the larger class to the size of the smaller class, to prevent bias in favor of the larger class during training. Next, both classes were randomly down-sampled to 90% of the size of the smaller class; this ensured that, in models where the class sizes were very similar at the outset, there was still variation in the data that went into each classifier. Finally, the classifier was trained and tested. This down-sampling procedure was repeated 500 times, yielding 500 different versions of the same classifier type. Mean performance metrics across all re-samplings were then compared between the two models.

## References

[1] A. J. Calder, “Facial emotion recognition after bilateral amygdala damage: Differentially severe impairment of fear,” *Cogn. Neuropsychol.*, vol. 13, no. 5, pp. 699–745, Jul. 1996, doi: 10.1080/026432996381890.

[2] J. P. Newman and D. S. Kosson, “Passive avoidance learning in psychopathic and nonpsychopathic offenders,” *J. Abnorm. Psychol.*, vol. 95, no. 3, pp. 252–256, Aug. 1986.

[3] A. C.-N. Wong, T. J. Palmeri, and I. Gauthier, “Conditions for face-like expertise with objects: Becoming a Ziggerin expert - but which type?,” *Psychol. Sci.*, vol. 20, no. 9, pp. 1108–1117, Sep. 2009, doi: 10.1111/j.1467-9280.2009.02430.x.

[4] T. A. Hare, N. Tottenham, M. C. Davidson, G. H. Glover, and B. J. Casey, “Contributions of amygdala and striatal activity in emotion regulation,” *Biol. Psychiatry*, vol. 57, no. 6, pp. 624–632, Mar. 2005, doi: 10.1016/j.biopsych.2004.12.038.

[5] N. Tottenham, T. A. Hare, and B. J. Casey, “Behavioral assessment of emotion discrimination, emotion regulation, and cognitive control in childhood, adolescence, and adulthood,” *Dev. Psychol.*, vol. 2, p. 39, 2011, doi: 10.3389/fpsyg.2011.00039.

[6] K. P. Schulz, J. Fan, O. Magidina, D. J. Marks, B. Hahn, and J. M. Halperin, “Does the emotional go/no-go task really measure behavioral inhibition? Convergence with measures on a non-emotional analog,” *Arch. Clin. Neuropsychol. Off. J. Natl. Acad. Neuropsychol.*, vol. 22, no. 2, pp. 151–160, Feb. 2007, doi: 10.1016/j.acn.2006.12.001.

[7] G. Kohls *et al.*, “Investigating sex differences in emotion recognition, learning, and regulation among youths with conduct disorder,” *J. Am. Acad. Child Adolesc. Psychiatry*, vol. 59, no. 2, pp. 263–273, Feb. 2020, doi: 10.1016/j.jaac.2019.04.003.

[8] R. J. R. Blair, E. Leibenluft, and D. S. Pine, “Conduct disorder and callous-unemotional traits in youth,” *N. Engl. J. Med.*, vol. 371, no. 23, pp. 2207–2216, Dec. 2014, doi: 10.1056/NEJMra1315612.

[9] R. Palermo, K. B. O’Connor, J. M. Davis, J. Irons, and E. McKone, “New Tests to Measure Individual Differences in Matching and Labelling Facial Expressions of Emotion, and Their Association with Ability to Recognise Vocal Emotions and Facial Identity,” *PLoS ONE*, vol. 8, no. 6, Jun. 2013, doi: 10.1371/journal.pone.0068126.

[10] M. K. Epstein, N. G. Poythress, and K. O. Brandon, “The Self-Report Psychopathy Scale and passive avoidance learning: a validation study of race and gender effects,” *Assessment*, vol. 13, no. 2, pp. 197–207, Jun. 2006, doi: 10.1177/1073191105284992.
